# Left bundle branch pacing combined with atrioventricular node ablation in atrial fibrillation with severe aortic stenosis: A case report

**DOI:** 10.1097/MD.0000000000046784

**Published:** 2026-01-09

**Authors:** Shujie Zhang, Lujing Nie, Lifan Shao, Yang Zhang, Wenjiu Feng, Qing Yin, Yanbo Chen

**Affiliations:** a Department of Cardiology, The First Affiliated Hospital of Shandong Second Medical University, Weifang, Shandong Province, China; b Emergency Department, Affiliated Hospital of Shandong University of Traditional Chinese Medicine, Jinan, Shandong Province, China.

**Keywords:** atrial fibrillation, atrioventricular node ablation, case report, left bundle branch pacing

## Abstract

**Rationale:**

In patients with atrial fibrillation (AF) complicated by severe aortic stenosis (AS), atrioventricular node ablation (AVNA) combined with permanent pacemaker implantation is an effective treatment strategy, especially when conventional rhythm control methods fail.

**Patient concerns:**

An 88-year-old female with a 40-year history of paroxysmal palpitations and chest tightness, with significant worsening of symptoms over the past 20 days. Despite undergoing AF ablation 5 years ago, her symptoms and arrhythmia burden persisted.

**Diagnosis:**

Long-standing (24 years) AF, severe AS, and a history of failed radiofrequency catheter ablation for AF.

**Interventions:**

The patient underwent left bundle branch pacing followed by AVNA.

**Outcomes:**

During the 9-month follow-up period, her palpitations completely resolved, with no recorded atrial arrhythmias. The AF’s impact on quality of life score significantly improved, and her performance on the 6-minute walk test markedly increased, indicating significant functional enhancement.

**Lessons:**

This case demonstrates that left bundle branch pacing combined with AVNA is clinically effective and technically feasible for AF patients in the elderly population with severe AS, particularly when conventional rhythm control methods fail. This approach offers an effective option for symptom relief and functional improvement in this challenging patient population.

## 1. Introduction

The management of symptomatic atrial fibrillation (AF) in patients with severe aortic stenosis (AS) remains a complex clinical dilemma, particularly since rhythm control strategies frequently fail in the setting of advanced structural heart disease. In such cases, an “ablate and pace” approach provides definitive symptom relief when pharmacological rate control is ineffective. However, traditional right ventricular pacing (RVP) may exacerbate ventricular dyssynchrony, further impairing hemodynamics in an already pressure-overloaded heart. Left bundle branch pacing (LBBP), a novel conduction system pacing (CSP) technique, has demonstrated superior outcomes by preserving physiological ventricular activation. We 1st report the case of an elderly female with long-standing AF and severe AS who was successfully treated with LBBP combined with atrioventricular node ablation (AVNA) after previous failed ablation.

## 2. Case presentation

**Figure 1.**
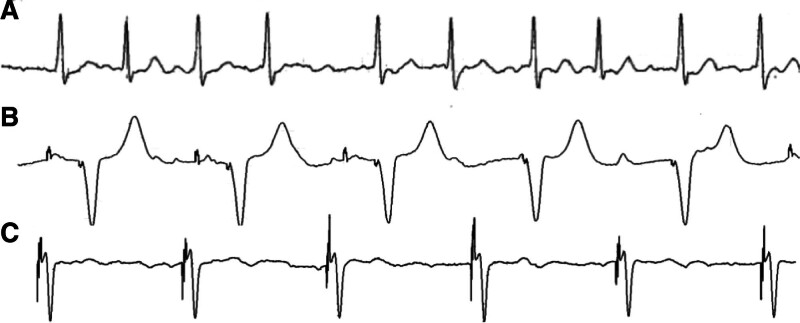
Electrocardiogram: (A) baseline atrial fibrillation. (B) Typical right ventricular pacing (QRSd = 163 ms). (C) Post-LBBaP (QRSd = 108 ms). LBBaP = left bundle branch area pacing, QRSd = QRS duration.

This 88-year-old female patient presented to our hospital on September 25, 2024, with a chief complaint of paroxysmal palpitations with notable chest oppression persisting for over 40 years and aggravated over the past 20 days. Forty years ago, she began to feel palpitations, and her pulse rate was approximately 180 beat/min. She was diagnosed with AF 20 years ago and underwent bilateral pulmonary vein isolation via radiofrequency catheter ablation 5 years ago, without significant improvement in either arrhythmia burden or symptom severity post-procedure. Physical examination revealed: no precordial bulge, palpable apical impulse at the 5th intercostal space without heaving, heart rate approximately 70 bpm with irregular rhythm, and a systolic ejection murmur was heard at the 2nd right intercostal space of the sternum, accompanied by diminished 2nd heart sound. Medical history includes: 60-year history of hypertension managed with regular amlodipine besylate and a surgery history of thyroid carcinoma. Diagnostic workup showed: echocardiography revealed a heavily calcified aortic valve with severe stenosis, as quantified by an aortic valve area of 0.7 cm², a mean pressure gradient of 67 mm Hg, and a peak aortic jet velocity of 5.1 m/s. Other findings included left atrial enlargement (anteroposterior diameter 42.6 mm) and a preserved ejection fraction of 69%. Electrocardiogram demonstrated AF with rapid ventricular rate and ST-T changes (Fig. 1A). Laboratory findings included fibrinogen 4.44 g/L and BNP 277.33 pg/mL. Preoperative diagnosis included: AF, aortic valve stenosis and hypertension (grade 3, very high risk).

Upon admission, the patient expressed a strong desire for a definitive treatment to manage her symptoms, while explicitly stating her wish to avoid any future AF ablations, given a history of a suboptimal outcome from a radiofrequency ablation 5 years prior. Given the patient’s advanced age, long-standing AF, and history of a failed ablation, a repeat rhythm control strategy was initially excluded. Initial evaluation confirmed severe AS, for which she met the indications for transcatheter aortic valve replacement (TAVR) as a means to improve her long-term prognosis.^[1]^ However, after a thorough discussion of the potential risks and benefits, the patient made an informed decision to decline the TAVR procedure. Her firm declination of TAVR necessitated a paradigm shift in our therapeutic approach towards palliative management of her refractory AF symptoms. In this context, an “ablate and pace” strategy was identified as the most appropriate course of action. The pivotal element of this decision was the selection of the pacing method. Given the patient’s severe AS, conventional RVP was considered high-risk, as the resulting dyssynchrony would likely worsen cardiac function. Consequently, we chose LBBP to provide physiological activation, thereby mitigating the risk of pacing-induced cardiomyopathy.

**Figure 2.**
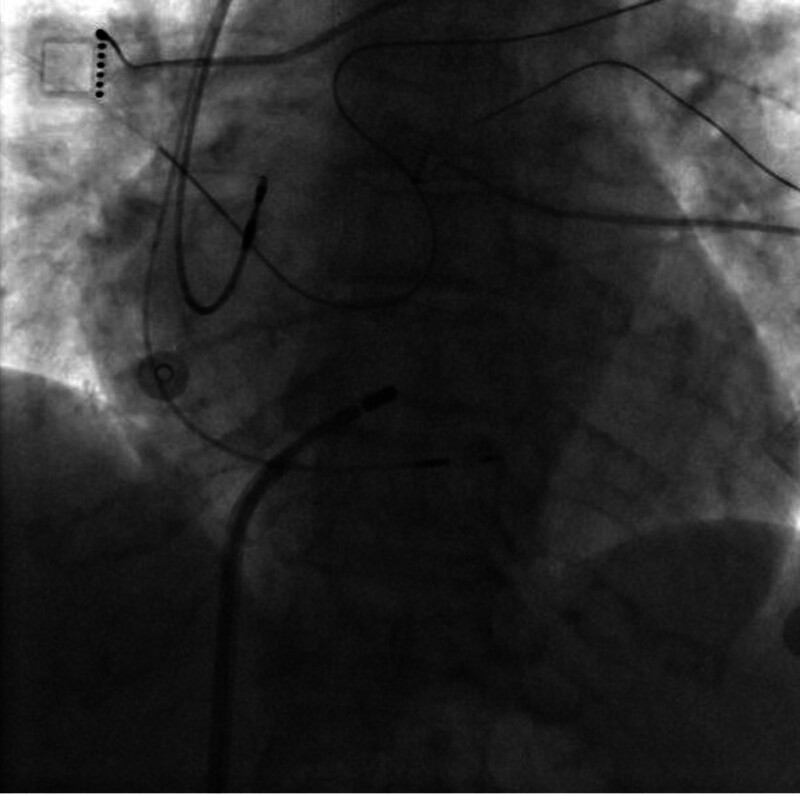
The anatomical relationship between the atrioventricular node ablation site and the left bundle branch pacing lead tip, as visualized through fluoroscopic imaging in the 30° right anterior oblique projection.

**Figure 3.**
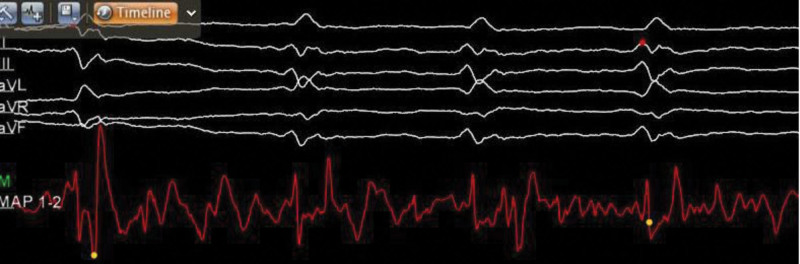
Intraoperative real-time electrophysiological mapping demonstrates successful atrioventricular node ablation.

The patient underwent a 2-stage procedure, beginning with the implantation of a dual-chamber left bundle branch area pacemaker, followed by AVNA. First, the left bundle branch pacemaker implantation was performed. The procedural steps were as follows. The patient was placed in a supine position, and the left subclavian vein was successfully punctured. A ventricular lead (Medtronic 3830–69 cm) was advanced to the left bundle branch area via an 8F peel-away sheath with the assistance of a C315 HIS sheath. Testing revealed a ventricular pacing threshold of 1.0 V, impedance of 670 ohms, and R-wave amplitude of 9 mV. An atrial lead (Abbott 2088TC-52 cm) was then positioned in the right atrial appendage, with test results showing an atrial pacing threshold of 0.8 V, impedance of 720 ohms, and P-wave amplitude of 2 mV. The leads were subsequently connected to the pulse generator (Abbott PM2172), confirming successful pacemaker implantation (Fig. 2). Following successful implantation of the left bundle branch area pacing (LBBaP) lead, the paced QRS duration was notably narrowed to 108 ms (Fig. 1C), indicating excellent capture of the left bundle branch area. This is in sharp contrast to the wide, left bundle branch block-like morphology typically seen with conventional RVP (typical QRS duration > 160 ms, Fig. 1B). Next, AV node radiofrequency ablation was performed under DSA. The patient remained in a supine position, and the right femoral vein was punctured to introduce a 3.5 mm D-curve ST ablation catheter. Using the CARTO3 mapping system, ablation was conducted at 40 to 45 W in power-controlled mode with saline irrigation at 30 mL/min, successfully ablating the AV node (Fig. 3). Postoperatively, the patient experienced no significant discomfort. She remained supine for 8 hours, and the wound was redressed after 24 hours. She was discharged approximately 48 hours later with a prescription for rivaroxaban (10 mg daily) for anticoagulation therapy.

Post-procedure, the patient achieved a heart rate of approximately 70 beats per minute with marked symptomatic improvement. The 6-minute walk test distance increased from < 150 meters preoperatively to 300 meters post-intervention. During the 9-month follow-up, the patient reported complete resolution of palpitations.

**Figure 4.**
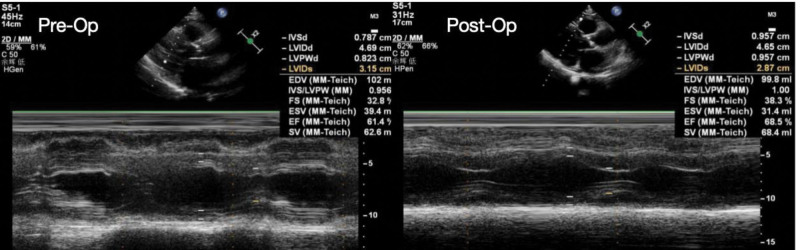
A comparison of pre- and post-operative echocardiograms demonstrated significant improvements in both cardiac structure and function: the left atrial diameter (LAD) decreased from 44.4 mm to 42.6 mm, the left ventricular end-diastolic diameter (LVIDd) reduced from 46.9 mm to 46.5 mm, while the ejection fraction (EF) increased from 61% to 69%, indicating markedly enhanced left ventricular systolic function and suggesting favorable therapeutic outcomes from the surgical intervention.

After 9 months of standardized treatment and regular follow-up, this patient with AF complicated by AS remained free from recurrent arrhythmias and achieved significant clinical improvement. The echocardiogram shows reduced atrial size and improved cardiac systolic function (Fig. 4). The patient reported marked symptomatic relief, specifically manifested as: substantial enhancement in daily activity tolerance: progressing from experiencing palpitations and dyspnea with mild exertion to being able to comfortably perform routine activities such as grocery shopping and walking; complete resolution of paroxysmal nocturnal dyspnea with consequent improvement in sleep quality; and noticeable reduction in exercise-induced fatigue.

Objective assessments demonstrated that the patient’s 6-minute walking distance improved from 410 meters to 523 meters, while the AFEQT quality of life score increased from a baseline of 52 (indicating severe impairment) to 78 (moderate impairment), consistent with the patient’s subjective perception of improvement. Throughout the follow-up period, the patient maintained excellent treatment adherence, showed significantly enhanced confidence in disease self-management, and attained substantial improvement in overall prognosis.

## 3. Discussion

Atrial fibrillation, the most prevalent clinical cardiac arrhythmia, is associated with poor clinical outcomes including reduced overall survival, and an increased risk of adverse events including stroke and heart failure.^[2]^ For many patients, AF causes distressing symptoms that dramatically affect daily living and wellbeing. When multiple risk factors coexist, the effectiveness of standard radiofrequency ablation and antiarrhythmic drug therapy for AF may be limited.^[3]^ The “pace-and-ablate” strategy is recommended as a superior option.

Due to the long-standing history of AF (lasting up to 20 years), the atrial substrate condition of the patient may be poor. Furthermore, she also presented with concomitant aortic valve stenosis, posing a more significant management challenge. Although it was possible to convert AF to sinus rhythm by radiofrequency ablation or electrical cardioversion, the recurrence rate of AF was likely to be high. Meanwhile, traditional pharmacological treatment was also predicted to yield suboptimal therapeutic outcomes coupled with substantial side effects. In such cases, the“pace-and-ablate” strategy appears to be more appropriate.^[4]^

The procedure involves ablating the AVN to block aberrant electrical conduction, while implanting a permanent pacemaker to establish a controlled ventricular rhythm.^[5]^ Compared to catheter ablation and direct current cardioversion, this approach serves as an effective alternative to avoid long-term antiarrhythmic drug therapy.^[6]^

Among all the pacing methods, CSP has emerged as the most widely utilized approach. Evidence confirms that CSP demonstrates superior preservation of left ventricular synchrony, closely resembling natural physiological activation patterns.^[7]^ Currently, CSP includes his bundle pacing and LBBP. When comparing these 2 strategies, his bundle pacing technique faces challenges such as difficulty in precise localization, suboptimal lead stability, and high procedural complexity owing to its deep location.^[8]^ In addition, LBBP can overcome the above shortcomings, characterized by optimal pacing parameters and superior safety performance. Due to its favorable efficacy and safety profile, LBBP has become the preferred CSP approach,^[4]^ especially the female patients.^[9]^

A key achievement in this case was the successful implementation of LBBP, which significantly narrowed the QRS duration of the paced waveform from the wide and desynchronized morphology of traditional RVP to 108 ms. LBBaP directly captures the left bundle branch conduction system, restoring a physiological ventricular activation sequence. This not only immediately optimizes cardiac pumping efficiency but also provides long-term protection for the patient, effectively preventing pacing-induced cardiomyopathy caused by ventricular dyssynchrony. Additionally, the procedure achieved a low and stable pacing threshold, further confirming the clinical value of LBBaP as an efficient and safe physiological pacing strategy.^[10]^

The successful outcome of this case carries significant clinical implications, establishing a novel therapeutic paradigm for this challenging patient population. For individuals with severe AS and refractory AF who are not candidates for or decline interventions like TAVR or repeat ablation, therapeutic options have traditionally been limited. Our findings suggest that CSP-enhanced “ablate and pace” therapy represents a viable and effective alternative. The key innovation lies in utilizing LBBP, which achieves dual benefits: ensuring consistent rate control while maintaining ventricular synchrony – a crucial consideration for ventricles already stressed by pressure overload from severe AS. Consequently, these findings support a paradigm shift in clinical practice: in managing symptomatic patients with severe AS and refractory AF, clinicians should prioritize the LBBP–AVNA combination as an initial palliative strategy, rather than reserving it as a last resort. By safeguarding cardiac function while optimizing life quality, this method addresses both physiological and psychosocial outcomes.

Several limitations should be acknowledged: the relatively brief follow-up period restricts longitudinal evaluation; and the single-case design limits extrapolation of results to broader clinical settings. Despite inherent study constraints, this case carries significant clinical implications. As the 1st reported successful application of dual-chamber pacemaker implantation combined with AVNA in treating long-standing persistent AF (AF duration > 20 years) complicated by valvular heart disease, this study establishes a novel device-ablation hybrid therapeutic paradigm for this clinically challenging population.

## 4. Conclusion

This case report demonstrates for the 1st time that LBBP combined with AVNA is a safe, technically feasible, and clinically effective therapeutic strategy for elderly patients with AF and severe AS, particularly after prior rhythm control strategies have failed. This approach not only ensures effective rate control and complete symptom resolution but also improves cardiac function and quality of life through physiological pacing, offering a valuable new option for this complex, high-risk patient population.

## Author contributions

**Conceptualization:** Shujie Zhang, Lujing Nie, Lifan Shao, Yang Zhang, Wenjiu Feng.

**Data curation:** Shujie Zhang, Lujing Nie, Qing Yin.

**Funding acquisition:** Yanbo Chen.

**Software:** Shujie Zhang, Lujing Nie.

**Writing – original draft:** Shujie Zhang, Lujing Nie, Lifan Shao, Yang Zhang, Wenjiu Feng.

**Writing – review & editing:** Yanbo Chen.
